# An Apoptosis-Related Gene Prognostic Index for Colon Cancer

**DOI:** 10.3389/fcell.2021.790878

**Published:** 2021-12-08

**Authors:** Hanmin Tang, Jing Wang, Xuehui Luo, Qi Wang, Jie Chen, Xinyue Zhang, Qiuting Li, Chengyi Gao, Yuesen Li, Suxia Han

**Affiliations:** Department of Radiation Oncology, First Affiliated Hospital of Xi’an Jiaotong University, Xi’an, China

**Keywords:** apoptosis gene, prognostic index, colon cancer, tumor microenvironment, treatment, nomograms

## Abstract

**Purpose:** To construct an apoptosis-related gene prognostic index (ARGPI) for colon cancer, and clarify the molecular and immune characteristics of the risk subgroup as defined by the prognostic index and the benefits of adjuvant chemotherapy. Integrating the prognostic index and clinicopathological risk factors to better evaluate the prognosis of patients with colon cancer.

**Methods:** Based on the colon adenocarcinoma data in the TCGA database, 20 apoptosis-related hub genes were screened by weighted gene co-expression network analysis (WGCNA). Five genes constituting the prognosis model were determined by Cox regression and verified by the Gene Expression Omnibus (GEO) dataset. Then the molecular and immune characteristics of risk subgroups defined by the prognostic index and the benefits of adjuvant chemotherapy were analyzed. Finally, nomograms integrating ARGPI and four clinicopathological risk factors were used to evaluate the prognosis of patients with colon cancer.

**Results:** The ARGPI was constructed based on the FAS, VWA5A, SPTBN2, PCK1, and TIMP1 genes. In the TCGA cohort, patients in the low-risk subgroup had a longer progression-free interval (PFI) than patients in the high-risk subgroup, which coincided with the results of the GEO cohort. The comprehensive results showed that the high-risk score was related to the enrichment of the cell cycle pathway, high mutation rate of TP53 and KRAS, high infiltration of T regulatory cells (Tregs), immunosuppressive state, and less chemotherapeutic benefit. However, low-risk scores are related to drug metabolism-related pathways, low TP53 and KRAS mutation rates, high infiltration of plasma cells, more resting CD4 memory cells and eosinophils, active immune function, and better chemotherapeutic benefits. Receiver operating characteristic curve of two-year progress prediction evaluation showed that the ARGPI had higher prognostic accuracy than TNM staging. Nomograms integrating ARGPI and clinicopathological risk factors can better evaluate the prognosis of patients with colon cancer.

**Conclusions:** The ARGPI is a promising biomarker for determining risk of colon cancer progression, molecular and immune characteristics, and chemotherapeutic benefit. This is a reliable method to predict the prognosis of colon cancer patients. It also can assist doctors in formulating more effective treatment strategies.

## Introduction

Globally, colorectal cancer is the third most common malignant tumor and second most common cause of death due to malignant tumors (Bray et al., 2018). At present, radical surgery remains the main treatment scheme for non-metastatic colon cancer. However, postoperative recurrence is a major problem. Approximately 80% of recurrence occurs in the first 3 years after treatment, often causing death (Sargent et al., 2005). For colon cancer with distant metastasis, even after receiving palliative treatment, the median survival time of patients is only 10–12 months (Labianca et al., 2010). Therefore, to better tumor control, it is essential to predict tumor recurrence and death due to colon cancer. At present, prognosis evaluation and treatment plan formulation of colon cancer patients largely depend on the TNM staging system (Kehoe and Khatri, 2006). However, due to tumor heterogeneity, even with the same tumor stage, there are still huge differences in disease progression and clinical results. This suggests the limitation of the TNM staging system in predicting the prognosis of patients with colon cancer. Therefore, it is necessary to develop new and reliable biomarkers to accurately predict the prognosis of patients with colon cancer.

Apoptosis is a programmed cell death regulated by genes and plays a very important role in maintaining human homeostasis and normal function (Rich et al., 1999). Apoptosis triggers cell death by activating cysteine protease, resulting in morphological changes in nuclear concentration and membrane vesiculation (Galluzzi et al., 2018). If DNA damaged cells do not receive the signal of apoptosis, then the balance between cell division and cell death may lead to cancer (Fan and Guo 2001). Studies have shown that inhibition of apoptosis is involved in the initial stage of carcinogenesis. For example, the combination of Fas and FasL can start the transduction of apoptosis signals and cause apoptosis. In the process of tumor development, Fas (CD95) is often downregulated, and tumor cells become anti-apoptotic (Fouqué et al., 2014). In addition, an important mechanism of chemoresistance is to inhibit tumor cell apoptosis. For example, Bcl-2 anti-apoptotic molecules are frequently upregulated in acquired drug-resistant cancer cells to block drug-induced apoptosis (Maji et al., 2017). However, there are few studies on the relationship between apoptosis and prognosis of colon cancer using high-throughput expression profiling.

In this study, we used weighted gene co-expression network analysis (WGCNA) and Cox regression to construct the apoptosis-related gene prognosis index (ARGPI), which improves risk stratification of colon cancer prognosis. The nomogram, based on progression-free interval (PFI), integrates the ARGPI and four clinicopathological risk factors, which are used to evaluate the clinical prognosis.

## Materials and Methods

### Patients and Datasets

The colon adenocarcinoma-associated mRNA sequences and clinical data used in this study were from TCGA^1^ and GEO (GSE39582) databases. Patients who met the following selection criteria were included: (A) the primary site of the tumor must be the colon, not colon metastasis; (B) The pathological type of colon cancer must be adenocarcinoma; (C) Available gene expression data; (D) Available survival information. The clinical data details of two train datasets used in this study are shown in Supplementary Table S1. The list of apoptosis-related genes was extracted from molecular signatures database^2^ (Liberzon et al., 2015). The search strategy includes the following keywords: “apoptosis” and “*Homo sapiens*.” Finally, 2,737 apoptosis-related genes were listed. PFI (progression-free interval), the most reliable prognostic index recommended in the TCGA database, was selected as the end point of disease progression. PFI is the period from the date of diagnosis until the date of the first occurrence of a new tumor event, which includes progression of the disease, locoregional recurrence, distant metastasis, new primary tumor, or death with tumor (Liu J. et al., 2018). Supplementary Figure S1 showed the flowchart for the present research.

### Identification of Apoptosis Related Hub Gene

The differentially expressed genes (DEGs) between colon cancer samples (379 cases) and normal samples (39 cases) in the TCGA dataset were identified using the limma package in R (*p*-value < 0.05, |log2FC| >1) (Ritchie et al., 2015). The apoptosis-related genes obtained from the molecular signatures database were intersected with the DEGs of colon cancer to obtain apoptosis-related DEGs. The DEGs related to apoptosis were analyzed by Gene Ontology (GO) and Kyoto Encyclopedia of Genes and Genomes (KEGG) using Metascape^3^ to determine the potential molecular mechanism of gene expression profile (Zhou et al., 2019).

Weighted gene co-expression network analysis (WGCNA) is a bioinformatics analysis method used to describe the gene association patterns between different samples (Langfelder and Horvath, 2008). Clustering of genes with similar expression patterns promotes web-based hub gene screening, which can be used to identify candidate biomarkers (Tang J. et al., 2018; Chen et al., 2021). The DEGs related to apoptosis were analyzed by the WGCNA package in R. First, the Pearson correlation was calculated from the expression data, and the relationship matrix was established. Then, the correlation matrix was transformed into an adjacency matrix, and the power exponential weighting was introduced to construct the scale-free network. Next, based on the adjacency matrix, the Tom matrix was established to calculate the Tom difference degree (1-TOM) between genes, and then the gene module was established. Finally, the results of the module were visualized, and the target module was selected for analysis. In this study, 127 genes of the most significantly related module (Turquoise module) were selected for further exploration. Twenty apoptosis-related hub genes significantly related to progression were screened by the R package (“survival” and “surviviner”) for further analysis.

### Construction and Validation of Apoptosis-Related Gene Prognostic Index

From 20 apoptosis-related hub genes, the genes independently related to the prognosis of colon cancer were screened by multivariate Cox regression analysis. The prognostic index of apoptosis related genes was determined using the following formula: ARGPI = Expression gene 1 * Coefficient + Expression gene 2 * Coefficient + + Expression gene * coefficient. The value of the ARGPI was defined as the risk score of each patient. According to the median risk score, the patients in the two databases were divided into the high-risk group and low-risk group for follow-up evaluation. The prognostic evaluation ability of the ARGPI was evaluated by plotting the K-M survival curve of TCGA and GEO cohort (*p* < 0.05, log rank test).

### Analysis of Molecular Characteristics of Different ARGPI Subgroups

Gene set enrichment analysis (GSEA) is a method to evaluate microarray data at the level of gene set (Subramanian et al., 2005). In the GSEA software, the gene set (c2. cp.kegg.v7.4. symbols.gmt) was used as the reference gene set to explore the important signal pathways enriched in high-risk subgroups and low-risk subgroups (*p* < 0.05 and FDR <0.25). In the analysis of gene mutation, information on mutations related to colon cancer samples was obtained from the TCGA database. Somatic variants in tumor patients in the high-risk and low-risk subgroups were comprehensively analyzed using the Maftools package in R (Mayakonda et al., 2018).

### Analysis of Immune Characteristics of Different ARGPI Subsets

CIBERSORT^4^ is a tool for deconvoluting the expression matrix of immune cell subtypes based on the principle of linear support vector regression. This method utilized expression profile data of colon cancer to estimate the infiltration of 22 types of immune cells in different risk subgroups (Newman et al., 2015). ssGSEA (single-sample gene set enrichment analysis) is used to study the enrichment level of immune-related functions in tissue expression profiles (Barbie et al., 2009). The enrichment levels of 29 immune-related functional features in different risk subgroups were analyzed using the GSVA package in R.

### Construction of Nomograms

To improve the prognostic risk stratification of colon cancer and assist in clinical diagnosis and treatment, a nomogram model integrating the ARGPI and clinicopathological features was constructed. It is used as a quantitative tool to predict the prognosis of patients with colon cancer. The effectiveness of the model was evaluated using a calibration curve.

### Statistical Analysis

Differences among variables were analyzed using an independent *t*-test and a chi-squared test. Univariate survival was assessed using Kaplan–Meier survival analysis and log rank test. Univariate Cox regression and multivariate Cox regression were used to analyze the effects of the ARGPI and clinical characteristics on prognosis. The data were statistically analyzed by R (version 4.1.1) and SPSS software (version 25.0). Bilateral *p* < 0.05 was considered statistically significant.

## Results

### The Apoptosis Related Hub Gene was Identified by WGCNA Analysis

**FIGURE 1:**
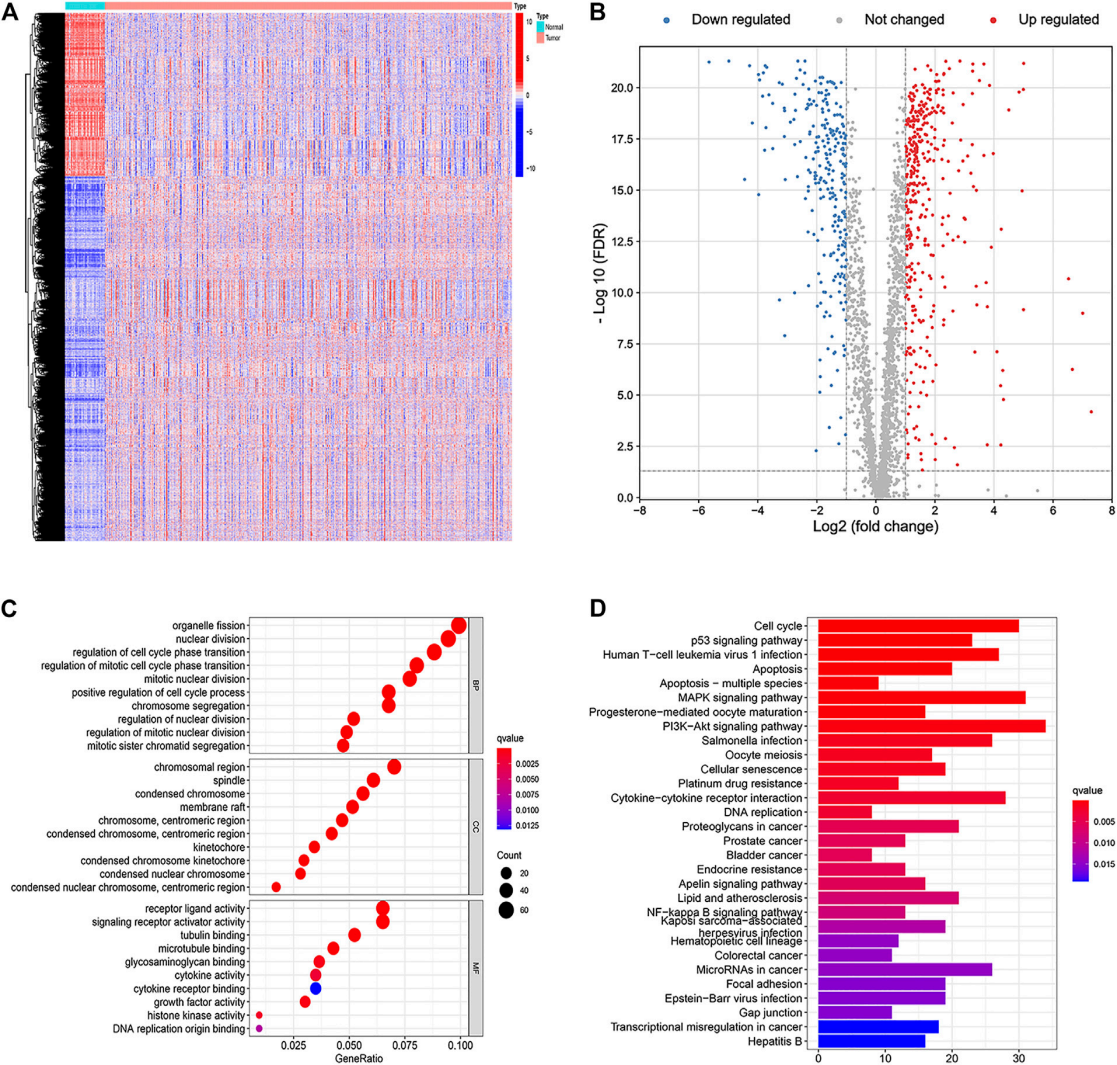
Identification and functional enrichment analyses of apoptosis-related differentially expressed genes. **(A)** Heat map of differentially expressed genes in colon cancer. **(B)** Volcano plot of apoptosis-related DEGs. **(C)** Gene Ontology (GO) enrichment analysis of the apoptosis-related DEGs. **(** **D** **)** Kyoto Encyclopedia of Genes and Genomes (KEGG) pathway analysis of the apoptosis-related DEGs.

A total of 7,859 DEGs were obtained by comparing colon adenocarcinoma samples with normal tissue samples, of which 5,432 genes were upregulated and 2,427 genes were downregulated (Figure 1A). Among these DEGs, 655 genes were related to apoptosis. Compared with normal samples, 392 genes were upregulated and 263 genes were downregulated in tumor samples (Figure 1B). Functional enrichment analysis identified GO terms and KEGG pathways that were enriched by 655 apoptosis-related DEGs (Figures 1C,D).

**FIGURE 2:**
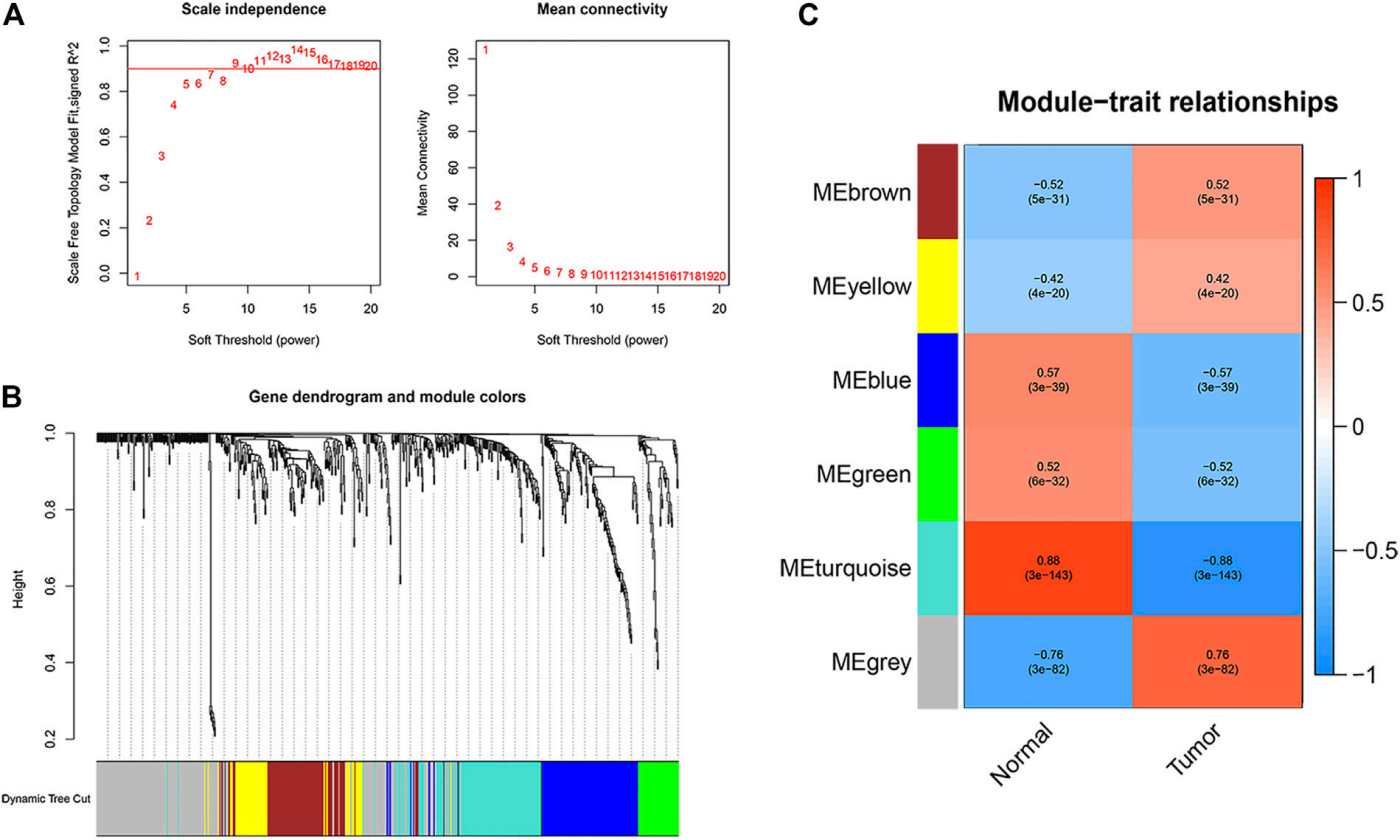
Identification of apoptosis related hub genes. **(A)** Determination of the soft-thresholding power in the WGCNA analysis. **(B)** Gene dendrogram and module colors. **(C)** Gene modules related to COAD obtained by WGCNA.

To identify apoptosis-related hub genes, WGCNA was used for candidate genes (n = 655). As shown in the left figure of Figure 2A, the horizontal axis is the soft threshold and the vertical axis is the evaluation parameters of scale-free network. The higher the value of evaluation parameters, the more consistent the network is with the characteristics of scale-free network. In the left graph, the horizontal line indicates that the threshold value is 0.90. As shown in the right figure of Figure 2A, the relationship between soft threshold (power) and mean connectivity shows that the optimal soft-thresholding power was seven based on the scale-free network. Finally, six modules are identified by hierarchical clustering and optimal soft threshold capability (Figure 2B). As shown in Figure 2C, the turquoise module with the highest absolute correlation with colon cancer was selected as the candidate module for further analysis, which contains 127 genes. Significantly enriched GO terms and KEGG pathways of turquoise modular genes are shown in Supplementary Figure S2. The expression profiles and clinical prognosis information of 127 genes in the turquoise module were obtained. Through K-M survival analysis, the expression of 20 apoptosis-related hub genes was closely related to the progression of colon cancer patients (Supplementary Figure S3; *p* < 0.05, log rank test).

### Prognostic Value of ARGPI in Colon Cancer

**FIGURE 3:**
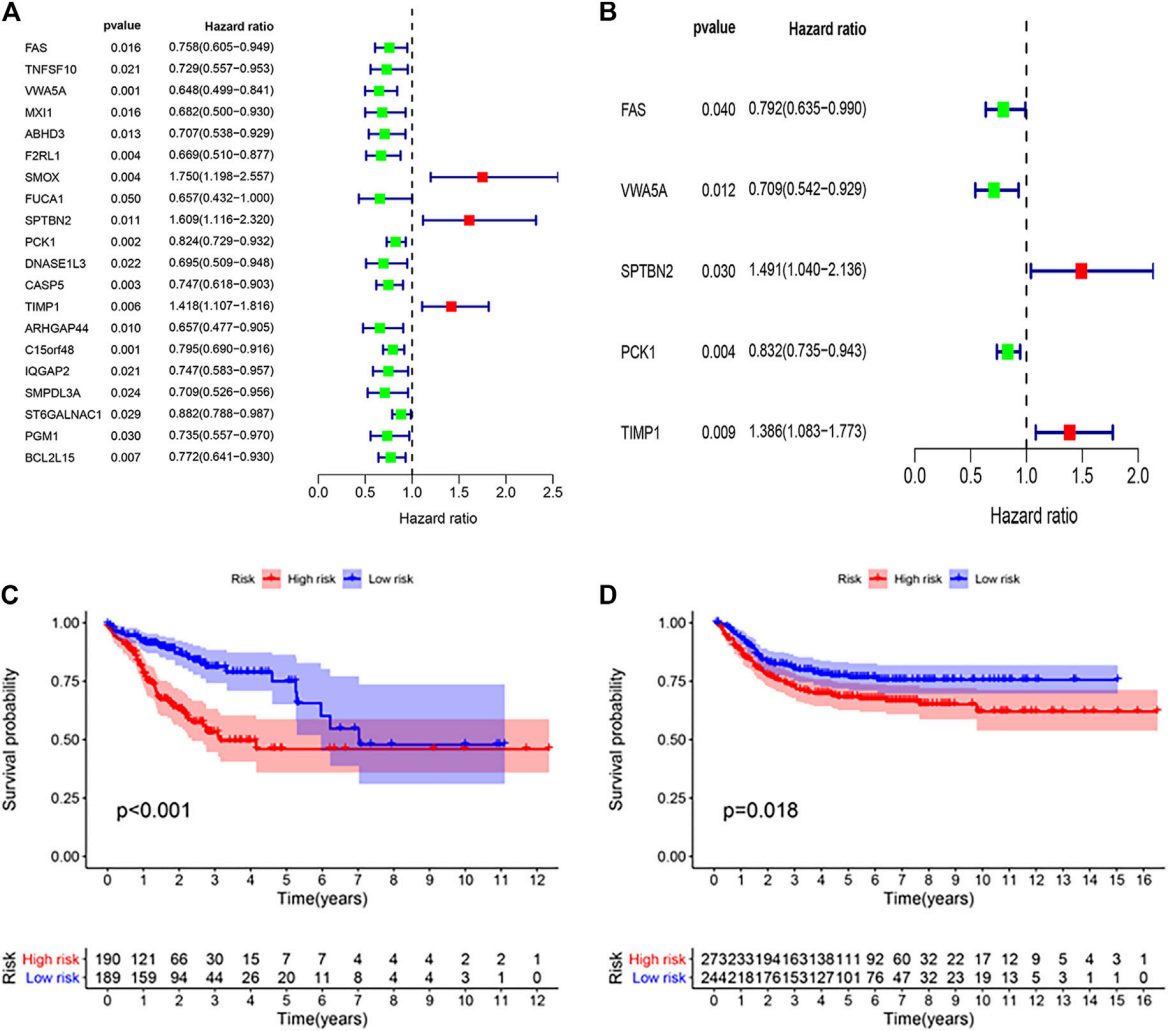
Prognostic analysis of different ARGPI subgroups. **(A)** Univariate Cox analysis of 20 apoptosis-related hub genes. **(B)** Apoptosis-related hub gene is independently associated with the prognosis of colon cancer. **(C)** Kaplan–Meier survival analysis of the ARGPI subgroups in the TCGA cohort. **(D)** Kaplan–Meier survival analysis of the ARGPI subgroups in the GEO cohort.

Multivariate Cox regression analysis was performed on 20 apoptosis-related hub genes to determine independent prognostic genes. As shown in Figures 3A,B, five genes (FAS, VWA5A, SPTBN2, PCK1, and TIMP1) significantly affect the prognosis of patients with colon cancer. Then, we constructed the ARGPI for all colon cancer patients. Through the Risk formula = Expression level of FAS * (−0.2326) + Expression level of VWA5A * (−0.3434) + Expression level of SPTBN2 * 0.3991 + Expression level of PCK1 * (−0.1833) + Expression level of TIMP1 * 0.3263. Taking the median risk value as the critical value, low-risk patients had better prognosis than high-risk patients (*p* < 0.001, log rank test) (Figure 3C). Then, the role of the prognostic model was verified by using the GSE39582 (n = 517) colon cancer data set. As shown in Figure 3D, the prognosis of patients in the low-risk group was significantly better than that in the high-risk group, which was concordant to the results of the TCGA dataset (*p* = 0.018, log rank test).

**FIGURE 4:**
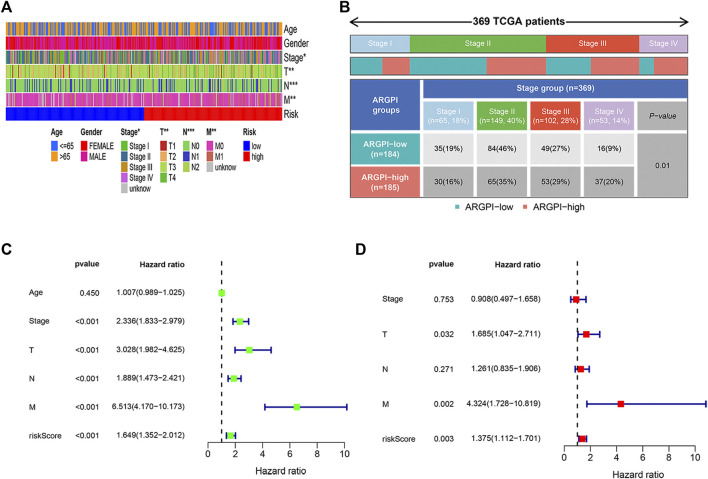
Clinical characteristics and independent prognosis of different ARGPI subgroups. **(A)** Distribution of clinical characteristics of ARGPI subgroup in TCGA cohort. **(B)** Differential distribution of tumor stage in ARGPI subgroup in TCGA cohort (*p* = 0.01). **(C)** Univariate Cox analysis of clinicopathological factors and the ARGPI score. **(D)** Independent prognostic analysis included clinicopathological factors and ARGPI score.

The distribution of clinicopathological features of 379 colon cancer patients in the TCGA cohort in different risk subgroups is shown in Figure 4A. In Figure 4B shows significant differences in the distribution of tumor stages between the two risk subgroups. Univariate Cox regression analysis showed that stage and risk score were significantly correlated to colon cancer prognosis (Figure 4C). Multivariate Cox regression analysis confirmed that risk score was an independent prognostic factor after adjusting for other clinicopathological factors (Figure 4D).

### Molecular Characteristics of Different ARGPI Subgroups

**FIGURE 5:**
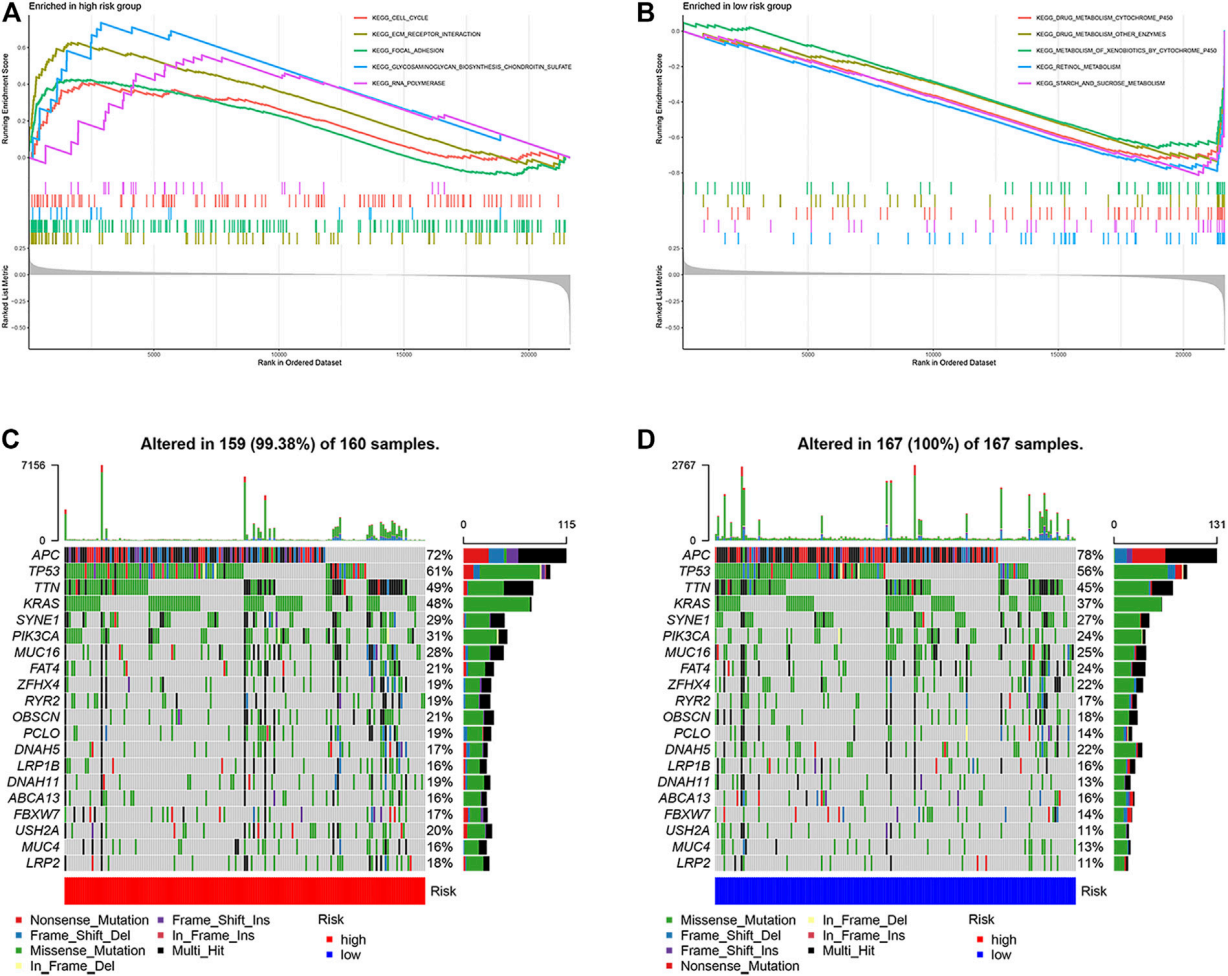
Molecular characteristics of different ARGPI subgroups. **(A)** Gene sets enriched in ARGPI-high subgroup. **(B)** Gene sets enriched in ARGPI-low subgroup. **(C)** Significantly mutated genes in the mutated COAD samples in ARGPI-high subgroup. **(D)** Significantly mutated genes in mutated COAD samples in ARGPI-low subgroup.

GSEA analysis was performed to identify pathways enriched in different risk subgroups. High-risk subgroup enriched CELL_CYCLE and FOCAL_ADHESION and other related pathways (Figure 5A). The low-risk subgroup was enriched in DRUG_ METABOLISM_ CYTOCHROME_ P450, RETINOL_ Metabolism and other related pathways (Figure 5B). Supplementary Table S2 lists the detailed results of GSEA, in which apoptosis related pathways are also enriched.

Next, we analyzed gene mutations to gain further biological insight into the nature of subgroup differences. We found that the mutation count in the low-risk subgroup was higher than that in the high-risk subgroup. Missense mutations were the most common mutation type, followed by nonsense mutations and frameshift deletions. We then identified the top 20 genes with the highest mutation rate in the risk subgroup (Figures 5C,D). The mutation rates of APC, TP53, TTN, KRAS, SYNE1, PIK3CA, MUC16 and FAT4 in the two groups were >20%.

### Immune Microenvironment of Different ARGPI Subgroups

**FIGURE 6:**
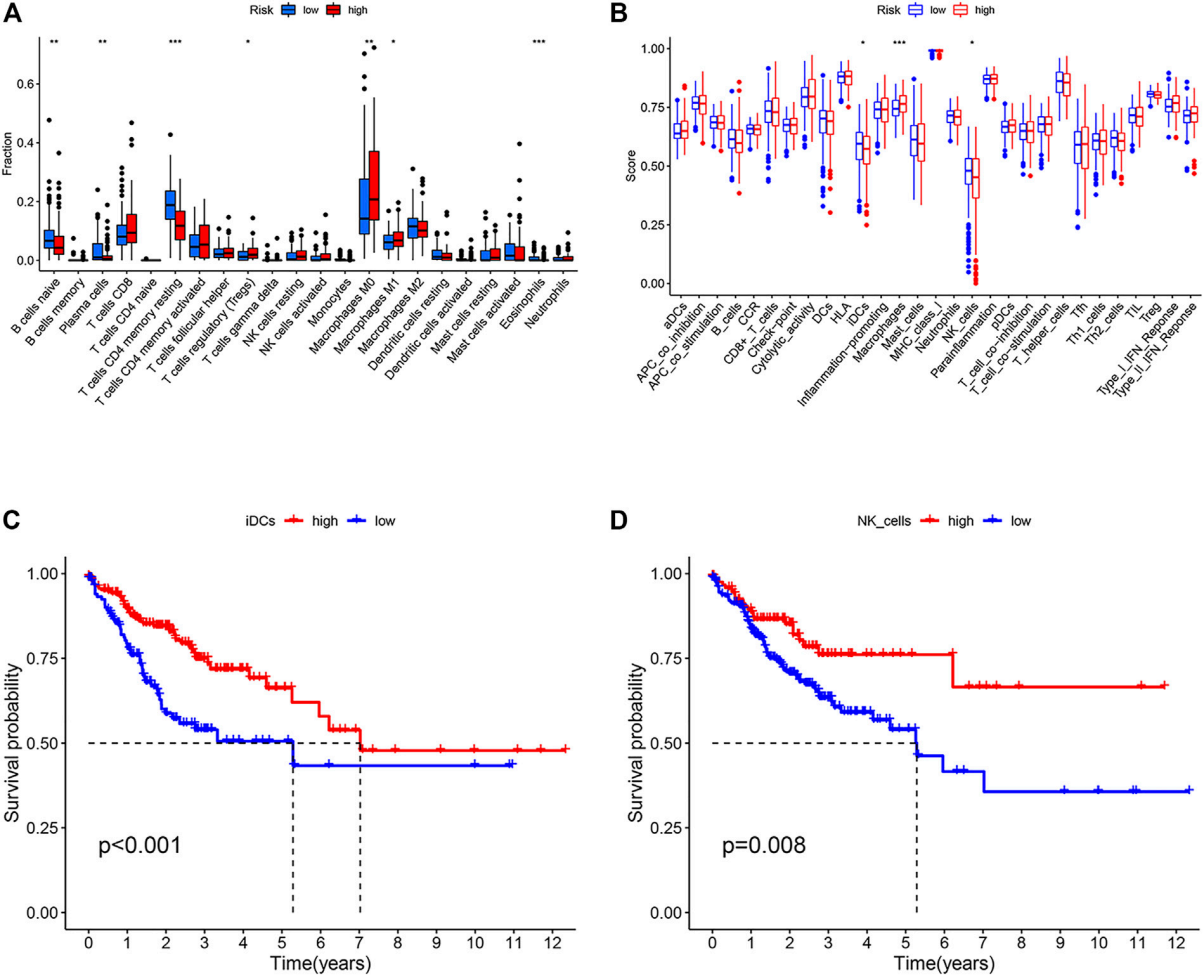
Immune characteristics of different ARGPI subgroups. **(A)** The proportions of the 22 immune cells in different ARGPI subgroups. **(B)** Immune related functions in different ARGPI subgroups. **(C)** Relationship between immune function and survival of interdigitating dendritic cells (IDCs). **(D)** Relationship between immune function and survival of NK cells.

To analyze the tumor microenvironment status in different risk subgroups, we first used CIBERSORT algorithm to estimate the proportion of 22 types of invasive immune cells in different risk subgroups. We found that macrophages M0, macrophages M1 and T cells regulatory (Tregs) were higher in the high-risk subgroup, while B cells naïve, plasma cells, T cells CD4 memory resting and eosinophils were more abundant in the low-risk subgroup (Figure 6A). We then applied the ssGSEA score to quantify the enrichment level of 29 immune features in each colon cancer sample. The results showed that there were more macrophages in the high-risk subgroup and more IDCs and NK_cells correlation signals in the low-risk subgroup (Figure 6B). We further studied the effects of immune and molecular functional differences between different risk subgroups on survival. As shown in Figures 6C,D, patients with more IDCs and NK_cells have better prognosis.

### Therapeutic Benefits of Different ARGPI Subgroups

**FIGURE 7:**
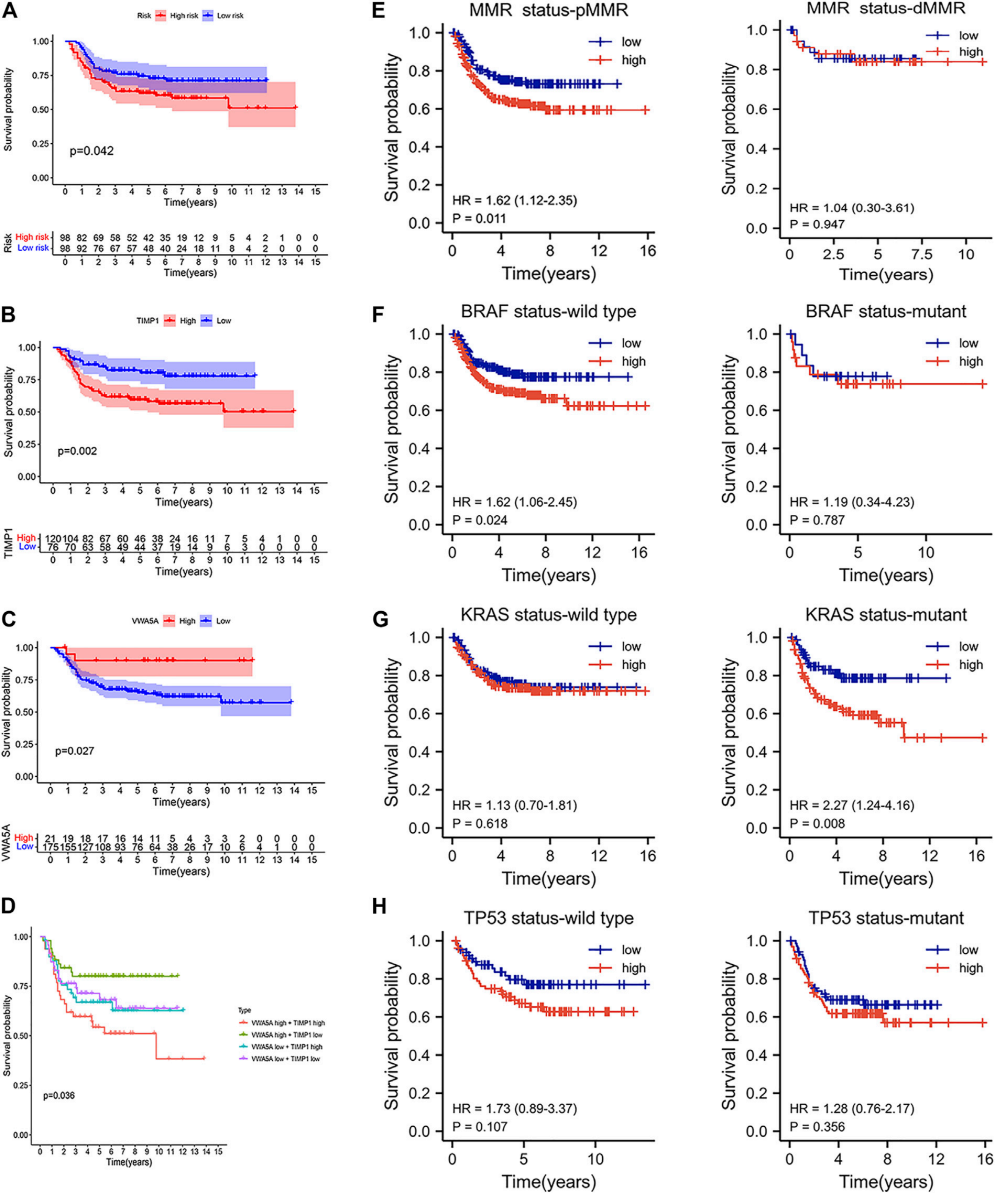
The benefit of therapy in different ARGPI subgroups. **(A)** Prognostic value of ARGPI in adjuvant chemotherapy of colon cancer. **(B)** Relationship between TIMP1 gene expression and the effect of adjuvant chemotherapy. **(C)** Relationship between VWA5A gene expression and the effect of adjuvant chemotherapy. **(D)** Double gene expression and the effect of adjuvant chemotherapy. **(** **E–H** **)** Effect of MMR/KRAS/BRAF/TP53 status on survival of different ARGPI subgroups.

To explore the relationship between apoptosis characteristics and treatment, we analyzed the chemotherapy data and molecular characteristics of colon cancer in the GEO (GSE39582) databases. As shown in Figure 7A, the high-risk group is more likely to progress after adjuvant chemotherapy than the low-risk group. Therefore, we observed the effect of the expression of five core genes on the progression after chemotherapy. As shown in Figures 7B,C, when the VWA5A gene is highly expressed, tumor patients make less progress after chemotherapy, suggesting that the upregulation of this gene promote chemotherapy sensitivity. When the TIMP1 gene is highly expressed, tumor patients progress more after chemotherapy, suggesting that the upregulation of TIMP1 gene reduce chemotherapy sensitivity. Figure 7D again shows that when the tumor shows upregulation of the VWA5A gene and downregulation of the TIMP1 gene, the tumor has better chemosensitivity. Stratified analysis showed that the apoptosis-related prognostic model was still a clinically and statistically significant prognostic model in patients with pMMR status, BRAF wild type, and KRAS mutation (Figures 7E–H).

### A Nomogram for Predicting the Prognosis of Patients with Colon Cancer

**FIGURE 8:**
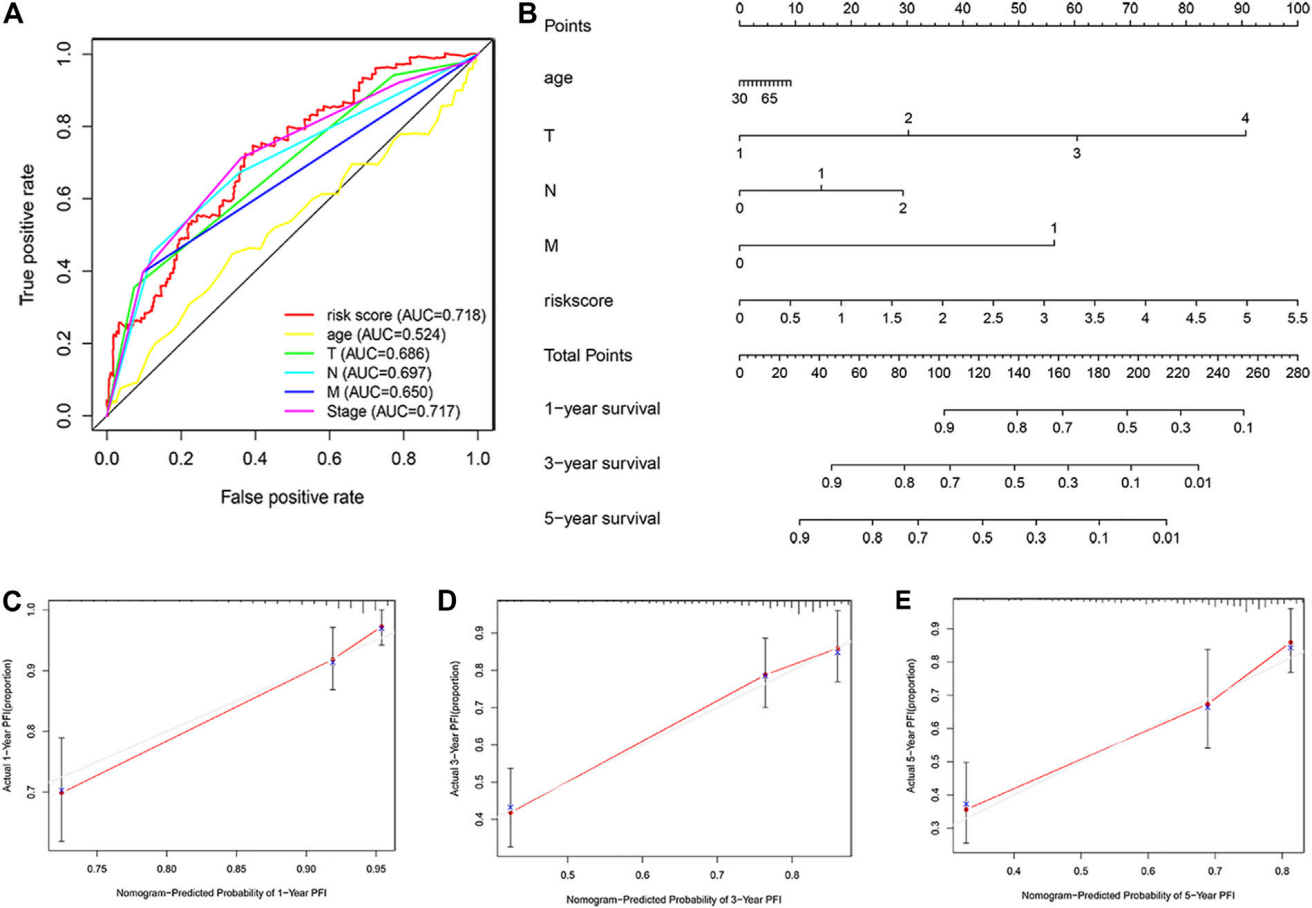
Nomograms for predicting colon cancer progression. **(A)** The receiver operating characteristic curve evaluates the accuracy of various indicators in predicting cancer progression within 2 years. **(B)** A nomogram predicts the risk of progression in patients with colon cancer by combining ARGPI and four clinicopathological features. **(** **C–E** **)** The calibration curve is used to evaluate the accuracy of one-, three-, and five-year progress forecasts of nomograms.

Receiver operating characteristic curve predicted by two-year progression showed that the ARGPI had higher prognostic accuracy than other clinicopathological features (Figure 8A). Then, to provide clinicians with a quantitative method to predict the possible risk of cancer progression, we constructed a nomogram based PFI, which integrates the ARGPI and four clinicopathological risk factors (Figure 8B). The prediction accuracy of the nomogram is as follows. The calibration curve of the nomogram shows that it does not deviate from the reference line and does not need to be recalibrated [Figure 8C (1 year), Figure 8D (3 years), Figure 8E (5 years)].

## Discussion

Colorectal cancer is the second most common cause of cancer death in the United States. According to the cancer statistics of the United States in 2021, it is estimated that there are about 149,500 patients with new colorectal cancer and about 52,980 patients who die from colorectal cancer (Siegel et al., 2021). Colon cancer is a malignant tumor that is easy to relapse after treatment, tumor progression is closely related to prognosis. At present, the depth of tumor invasion, regional lymph node invasion and distant metastasis (TNM stage) are still the most accurate prognostic tools. However, with the application of next-generation sequencing, molecular markers can be independent of TNM staging. Through risk stratification of colorectal cancer patients, the prediction of survival results has reached a high level of reliability (Yu et al., 2015). Although TNM staging remains the most convincing tool for us to evaluate the prognosis of patients and guide further treatment, the continuous expansion of our understanding of the molecular level in the occurrence and development of colon cancer allows improvement of staging and the prognosis system.

Apoptosis is an autonomous and orderly cell death controlled by genes to maintain the stability of the internal environment (Tower, 2015). Apoptosis is a complex process involving a large number of genes. The changes of these apoptotic genes play an important role in the formation, development, metastasis and drug resistance of malignant tumors (Fan and Guo, 2001; Wong, 2011). The role of apoptosis in the occurrence and development of colon cancer has been fully valued and studied. For example, changes in important molecules of key protein signaling pathways inducing apoptosis such as p53, APC, and RAS accumulate genetic errors, increasing the risk of colon cancer progression (Watson, 2006). In addition, changes in apoptosis pathways play an important role in anti-tumor therapy (such as radiotherapy and chemotherapy) (Potten and Grant 1998; Johnstone et al., 2002). These research results have prompted us to investigate apoptosis-related genes, their prospects as prognostic markers, and further provide new ideas for clinical treatment.

By combining the high-throughput expression profile of apoptosis-related genes and WGCNA analysis method that can effectively identify candidate biomarkers, we analyzed the prediction efficiency of apoptosis-related gene characteristics in predicting the prognosis of colon cancer patients and the potential impact on chemotherapy. Finally, the ARGPI was constructed based on five apoptosis genes (FAS, VWA5A, SPTBN2, PCK1, and TIMP1), which can effectively improve the prediction of colon cancer progression. In this model, both TCGA and GEO cohorts demonstrated that patient progression rate is higher in the high-risk subgroup and lower in the low-risk subgroup. In terms of its ability to predict progression, the predictive value of two-year progression is better than the classical TNM staging, which means that apoptosis signals can be used to improve the current prognostic evaluation system. Therefore, we combined the ARGPI and four clinicopathological risk variables (T, N, M, and age) to construct a nomogram to predict the probability of progression at one, three, and 5 years. This will help to effectively identify high-risk patients with progression and guide the formulation of more reasonable treatment schemes for colon cancer, which is effective for clinical application.

The ARGPI was comprised of five genes, FAS, VWA5A, SPTBN2, PCK1, and TIMP1. FAS (also known as TNFRSF6 or CD95) is a transmembrane protein. It is a cell surface receptor of a member of the tumor necrosis factor receptor superfamily. Its binding with FasL can start the transduction of apoptotic signals and cause apoptosis (Xiao et al., 2019). The expression of Fas in colorectal tissues of patients with colorectal cancer is often lower than in normal colorectal tissues. Studies have shown that the decrease in Fas expression may be the reason for the reduction in apoptosis in colon cancer cells (Mo et al., 2015). Silencing Fas expression promotes colon cancer immune escape and 5-fluorouracil resistance (Paschall et al., 2015). VWA5A (also known as BCSC-1 or LOH11CR2A), located on chromosome 11q23, contains two conserved domains, one of which is between the N-terminal vault proteins α-trypsin inhibitor domain, a C-terminal von Willebrand factor type a domain. Because it participates in the process of apoptosis induced by p53, it is considered as a potential tumor suppressor genes (Gentile et al., 2001). In breast cancer, BCSC-1 inhibits NF-κB signal transduction that disrupts breast cancer metastasis (Di et al., 2018). In nasopharyngeal carcinoma, the decrease of Wnt signal transduction may be involved in the tumor inhibition mechanism of BCSC-1 (Zhou et al., 2009). The main function of SPTBN2 (also known as GTRAP41 or SCAR14) is to regulate glutamate signaling pathway by stabilizing glutamate transporter EAAT4 on the plasma membrane surface. This gene mutation can lead to spinocerebellar ataxia (Yıldız et al., 2017). In ovarian cancer, the upregulation of SPTBN2 has been associated with poor prognosis (Feng et al., 2021). TIMP1, tissue inhibitor matrix metalloproteinase-1, belongs to the tissue inhibitor of metalloproteinases family. This protein can inhibit the proteolytic activity of matrix metalloproteinases (MMPs) and regulate the balance of matrix remodeling during extracellular matrix degradation (Batra et al., 2012). Studies have shown that TIMP1 is highly expressed in colon cancer and leads to tumor proliferation, metastasis, and anti-apoptosis through the FAK-PI3K/AKT and MAPK pathways (Song et al., 2016). A randomized controlled study showed that in patients with metastatic colorectal cancer receiving first-line combined chemotherapy, the upregulation of plasma TIMP-1 indicates that the objective effective rate of chemotherapy is very low (Sørensen et al., 2007). Studies have shown that the overexpression of TIMP-1 leads to reduced sensitivity to chemotherapy (Fu et al., 2011; Aaberg-Jessen et al., 2019). PCK1 (phosphoenolpyruvate carboxykinase 1) is a ladder-limiting enzyme of gluconeogenesis. Studies have shown that the expression level of PCK1 in liver cancer specimens and liver cancer cells is lower than that in normal samples. Overexpression of PCK1 can reduce the survival rate of liver cancer cell lines, induce apoptosis, inhibit cell migration, and activate the expression of PCK1 and thus may be potentially utilized as a therapeutic strategy for liver cancer (Liu MX. et al., 2018; Tang Y. et al., 2018).

Next, we analyzed gene mutations in different ARGPI subsets. Missense mutations, nonsense mutations, and frameshift deletion are the most common mutations in colon cancer, which is concordant to the reported tumor mutation load in colon cancer (Lee et al., 2017). In our study, the tumor mutation burden (TMB) was higher in the low-risk subgroup than the high-risk subgroup. High-TMB has been associated with better prognosis in colorectal cancer patients undergoing postoperative adjuvant chemotherapy (Lee et al., 2019). This may be one of the reasons for the observed lower recurrence rate after adjuvant chemotherapy in the low-risk and high-risk subgroup. The largest mutation difference between the two groups was observed in the KRAS, which was more common in high-risk samples than in low-risk subgroups (48 *vs*. 37%). Studies have shown that KRAS mutations may lead to poor prognosis by enhancing tumor cell proliferation (McCuaig et al., 2020). Clinical controlled studies have shown that the recurrence risk of KRAS mutant tumors is significantly higher than KRAS wild-type tumors (Hutchins et al., 2011). In our model, among patients with KRAS mutation, the low-risk subgroup made less progress than the high-risk subgroup.

Finally, we analyzed the tumor microenvironment of different ARGPI subsets. The composition of some immune cells in different ARGPI subsets varied. Macrophages M0 and M1 and T cells regulation (Tregs) were more abundant in the high-risk subgroup, while B cells naïve, plasma cells, T cells CD4 memory resetting and eosinophils were more common in the low-risk subgroup. Regulatory T cells (Tregs) play an important role in maintaining immune dynamic balance. However, studies have shown that inhibition of Treg function enhances antitumor effects (Yan et al., 2019). In addition, the activation of Wnt/-catenin signal in Tregs is related to the promotion of colon cancer (Keerthivasan et al., 2014). A large number of studies have shown that intensive infiltration of T cells, especially resting CD4 memory T cells, predicts good prognoses (Jin and Qin, 2020; Zhang et al., 2020). GSEA analysis showed that the high-risk subgroup was enriched with DEGs related to CELL CYCLE pathways, while low risk subgroups were enriched with DEGs associated with DRUG METABOLISM CYTOCHROME P450 pathways. This suggests that high-risk subgroups are characterized by tumor progression (Evan and Vousden, 2001). Most tumor chemotherapeutic drugs need to be achieved through CYP450 enzyme metabolism, and the low-risk subgroup may be more chemosensitive (Scripture et al., 2005).

## Conclusion

The ARGPI is a promising apoptosis related prognostic biomarker. The ARGPI, combined with TNM staging, may help distinguish high-risk groups, more accurately evaluate the prognosis of patients, and guide the formulation of more scientific treatment strategies. The ARGPI may also potentially used as a prognostic indicator of chemotherapy, although further research is warranted.

## Data Availability

The datasets presented in this study can be found in online repositories. The names of the repository/repositories and accession number(s) can be found in the article/Supplementary Material.

## Footnotes

1
https://portal.gdc.cancer.gov/

2
http://www.gsea-msigdb.org/gsea/msigdb/genesets.jsp

3
https://metascape.org

4
http://cibersort.stanford.edu/

## Abbreviations

ARGPI, apoptosis related gene prognostic index; COAD, colon adenocarcinoma; DEGs, differentially expressed genes; GEO, gene expression omnibus; GO, Gene Ontology; GSEA, gene set enrichment analysis; GSVA, gene set variation analysis; KEGG, Kyoto Encyclopedia of Genes and Genomes; PFI, progression free interval events; ROC, receiver operating characteristic curve; TCGA, the cancer genome atlas; TMB, tumor mutation burden.
