# Supplementary material for: An Apoptosis-Related Gene Prognostic Index for Colon Cancer
Source: Front Cell Dev Biol. 2021 Dec 8;9:790878. doi: 10.3389/fcell.2021.790878 (PMC8692577; doi:10.3389/fcell.2021.790878)
Supplement: Supplementary file 1 [file DataSheet1.PDF]

Supplementary Material

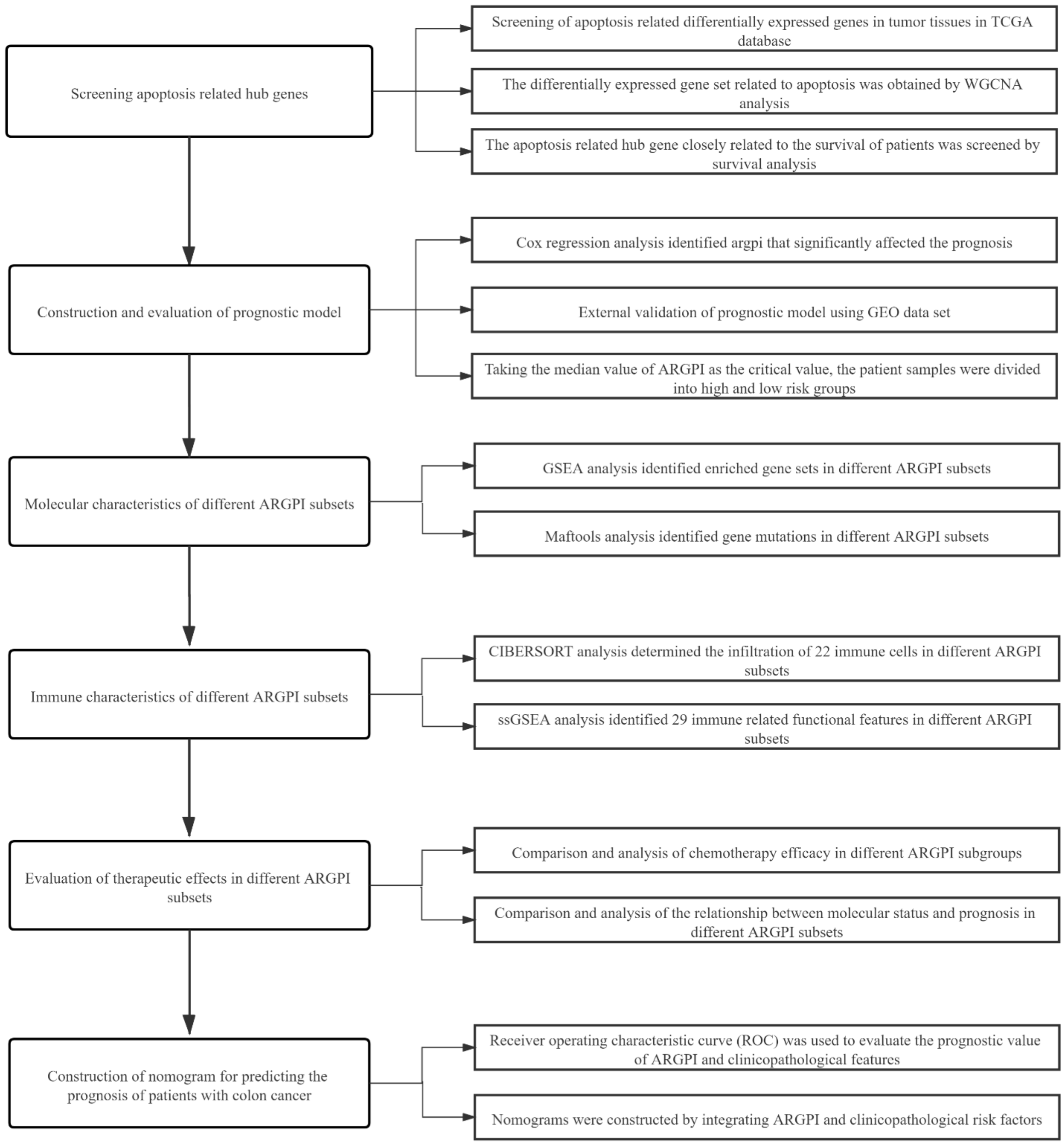

Supplementary Figure 1 | A flowchart summarizes the scheme for constructing the apoptosis related gene prognostic index (ARGPI) of colon cancer.

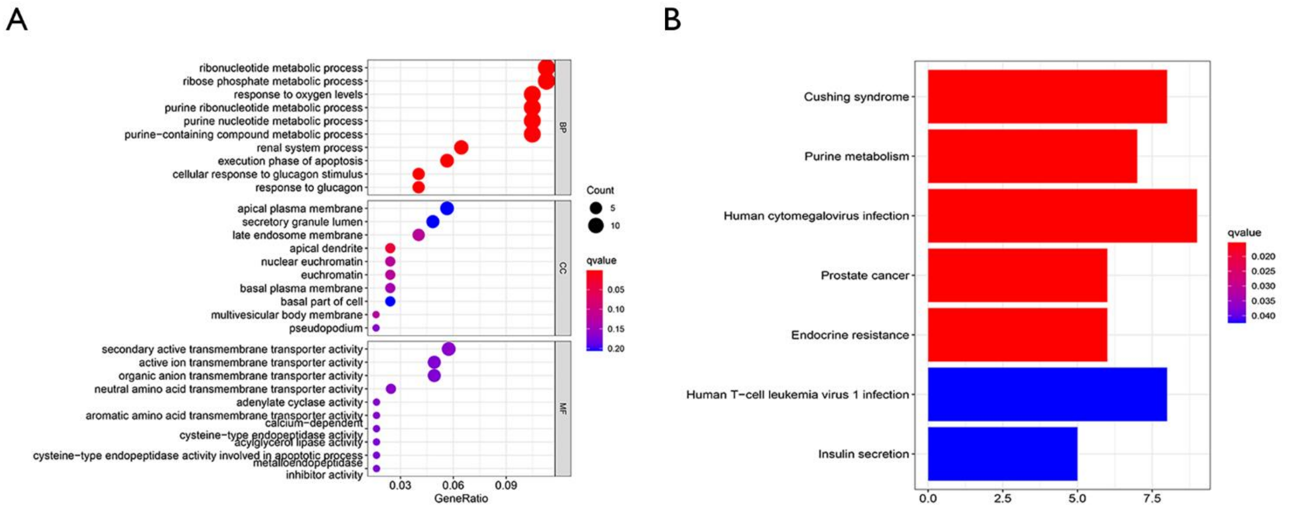

Supplementary Figure 2 | Functional enrichment analysis of turquoise module. (**A**) Gene Ontology (GO) enrichment analysis of the turquoise module. (**B**) Kyoto Encyclopedia of Genes and Genomes (KEGG) pathway analysis of the turquoise module.

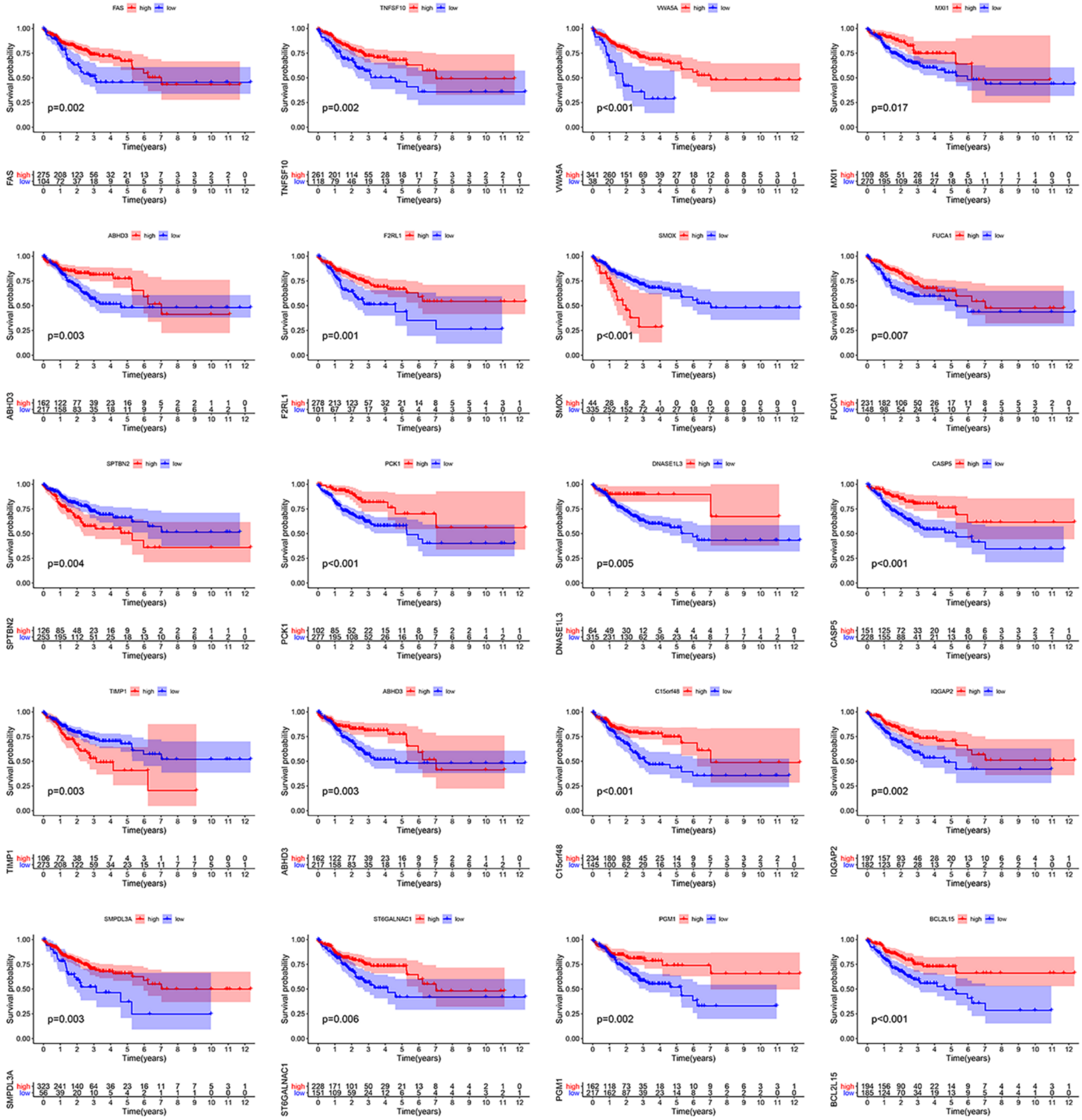

Supplementary Figure 3 | Kaplan-Meier survival analysis of 20 apoptosis-related genes in TCGA cohort.

Supplementary Table 1 | Baseline patient characteristic.

TABLE | Baseline patient characteristic.

Variable	Number (N)	
	TCGA cohort（379 patient）	GSE39582 cohort（517 patient）
Age		
<60	105	133
⩾60	274	384
Gender		
MALE	200	286
FEMALE	179	231
T		
T1	10	16
T2	67	45
T3	258	341
T4	44	97
NA	-	18
N		
N0	227	288
N1	87	122
N2	65	86
NA	-	21
M		
M0	316	469
M1	53	28
NA	10	20
Stage		
Stage I	65	36
Stage II	149	254
Stage III	102	194
Stage IV	53	28
NA	10	5

NA = not applicable.

Supplementary Table 2 | A list of gene sets enriched in different ARGPI subgroups in GSEA analysis.

TABLE | Gene sets enriched in different ARGPI subsets in GSEA analysis.

Description	setSize	enrichmentScore	NES	pvalue	p.adjust	qvalues
KEGG_ECM_RECEPTOR_INTERACTION	81	0.627245	2.470463	1.54E-10	2.83E-08	2.07E-08
KEGG_RETINOL_METABOLISM	45	-0.78988	-2.53598	4.31E-10	3.96E-08	2.90E-08
KEGG_STARCH_AND_SUCROSE_METABOLISM	36	-0.8129	-2.46537	1.27E-09	7.80E-08	5.71E-08
KEGG_DRUG_METABOLISM_CYTOCHROME_P450	58	-0.72269	-2.43791	1.52E-08	6.98E-07	5.11E-07
KEGG_FOCAL_ADHESION	194	0.426579	1.911466	4.83E-07	1.78E-05	1.30E-05
KEGG_DRUG_METABOLISM_OTHER_ENZYMES	37	-0.73598	-2.26315	2.57E-06	7.89E-05	5.78E-05
KEGG_GLYCOSAMINOGLYCAN_BIOSYNTHESIS_CHONDROITIN_SULFATE	22	0.735452	2.230031	4.97E-06	0.000127	9.28E-05
KEGG_METABOLISM_OF_XENOBIOTICS_BY_CYTOCHROME_P450	55	-0.66027	-2.21416	5.51E-06	0.000127	9.28E-05
KEGG_FATTY_ACID_METABOLISM	40	-0.72271	-2.26028	6.21E-06	0.000127	9.29E-05
KEGG_LINOLEIC_ACID_METABOLISM	23	-0.77545	-2.1218	2.03E-05	0.000373	0.000273
KEGG_PENTOSE_AND_GLUCURONATE_INTERCONVERSIONS	16	-0.82151	-2.05109	3.28E-05	0.000522	0.000382
KEGG_STEROID_HORMONE_BIOSYNTHESIS	41	-0.68127	-2.14259	3.43E-05	0.000522	0.000382
KEGG_PROXIMAL_TUBULE_BICARBONATE_RECLAMATION	22	-0.78221	-2.1243	3.69E-05	0.000522	0.000382
KEGG_ASCORBATE_AND_ALDARATE_METABOLISM	14	-0.84759	-2.04331	5.60E-05	0.000735	0.000539
KEGG_PEROXISOME	77	-0.57289	-2.01205	7.33E-05	0.000899	0.000658
KEGG_VALINE_LEUCINE_AND_ISOLEUCINE_DEGRADATION	43	-0.64134	-2.04116	0.000257	0.002953	0.002162
KEGG_PORPHYRIN_AND_CHLOROPHYLL_METABOLISM	28	-0.69767	-1.98373	0.000407	0.00441	0.003229

KEGG_CELL_CYCLE	122	0.407057	1.71614	0.000497	0.005081	0.003721
KEGG_PPAR_SIGNALING_PATHWAY	66	-0.54921	-1.9035	0.000636	0.006161	0.004512
KEGG_NITROGEN_METABOLISM	22	-0.70954	-1.92696	0.000773	0.006978	0.00511
KEGG_PROPANOATE_METABOLISM	31	-0.6547	-1.9222	0.000805	0.006978	0.00511
KEGG_BUTANOATE_METABOLISM	33	-0.6485	-1.92978	0.000834	0.006978	0.00511
KEGG_APOPTOSIS	87	-0.49297	-1.76523	0.001444	0.011553	0.00846
KEGG_GLYCOLYSIS_GLUCONEOGENESIS	61	-0.54268	-1.86298	0.0016	0.011917	0.008726
KEGG_OLFACTORY_TRANSDUCTION	108	-0.46251	-1.71603	0.001619	0.011917	0.008726
KEGG_ALDOSTERONE_REGULATED_SODIUM_REABSORPTION	41	-0.57768	-1.81681	0.003028	0.021432	0.015694
KEGG_INTESTINAL_IMMUNE_NETWORK_FOR_IGA_PRODUCTION	44	-0.55565	-1.77238	0.006067	0.041343	0.030274
KEGG_RNA_POLYMERASE	27	0.558711	1.7632	0.006839	0.04346	0.031825
KEGG_CITRATE_CYCLE_TCA_CYCLE	30	-0.59722	-1.73589	0.006893	0.04346	0.031825
KEGG_FRUCTOSE_AND_MANNOSE_METABOLISM	31	-0.57842	-1.69825	0.007086	0.04346	0.031825
KEGG_ETHER_LIPID_METABOLISM	25	-0.61256	-1.71509	0.008446	0.050132	0.03671
KEGG_TYROSINE_METABOLISM	40	-0.53052	-1.65921	0.011347	0.065245	0.047777
KEGG_HYPERTROPHIC_CARDIOMYOPATHY_HCM	81	0.381474	1.502471	0.013265	0.072674	0.053217
KEGG_DNA_REPLICATION	36	0.497862	1.676775	0.013763	0.072674	0.053217
KEGG_DILATED_CARDIOMYOPATHY	88	0.369673	1.4701	0.013824	0.072674	0.053217
KEGG_B_CELL_RECEPTOR_SIGNALING_PATHWAY	75	-0.45261	-1.58424	0.014231	0.072736	0.053262
KEGG_PYRUVATE_METABOLISM	39	-0.54174	-1.68	0.015119	0.073403	0.05375

KEGG_O_GLYCAN_BIOSYNTHESIS	28	-0.5743	-1.63294	0.015326	0.073403	0.05375
KEGG_INOSITOL_PHOSPHATE_METABOLISM	54	-0.48377	-1.60606	0.015558	0.073403	0.05375
KEGG_HEMATOPOIETIC_CELL_LINEAGE	83	-0.42971	-1.52584	0.016047	0.073815	0.054052
KEGG_ARACHIDONIC_ACID_METABOLISM	50	-0.50298	-1.65218	0.019075	0.085605	0.062685
KEGG_INSULIN_SIGNALING_PATHWAY	134	-0.38967	-1.48689	0.019877	0.087081	0.063766
KEGG_NICOTINATE_AND_NICOTINAMIDE_METABOLISM	23	-0.59679	-1.63297	0.020439	0.08746	0.064044
KEGG_ADIPOCYTOKINE_SIGNALING_PATHWAY	66	-0.44747	-1.55089	0.021599	0.088834	0.06505
KEGG_ABC_TRANSPORTERS	42	-0.50874	-1.60739	0.021726	0.088834	0.06505
KEGG_CYTOKINE_CYTOKINE_RECEPTOR_INTERACTION	250	-0.32618	-1.34753	0.022594	0.090377	0.06618
KEGG_STEROID_BIOSYNTHESIS	16	0.593487	1.640434	0.025037	0.098017	0.071775
KEGG_LONG_TERM_POTENTIATION	68	-0.43593	-1.51982	0.02858	0.109558	0.080226
KEGG_BASE_EXCISION_REPAIR	32	0.466869	1.51555	0.029823	0.111172	0.081408
KEGG_ALZHEIMERS_DISEASE	138	-0.3663	-1.41059	0.03021	0.111172	0.081408
KEGG_GNRH_SIGNALING_PATHWAY	92	-0.40775	-1.47406	0.032304	0.116547	0.085344
KEGG_TERPENOID_BACKBONE_BIOSYNTHESIS	15	-0.65083	-1.59844	0.033053	0.116957	0.085643
KEGG_T_CELL_RECEPTOR_SIGNALING_PATHWAY	107	-0.38139	-1.41301	0.034533	0.119887	0.087789
KEGG_PHOSPHATIDYLINOSITOL_SIGNALING_SYSTEM	74	-0.40945	-1.43541	0.040519	0.138064	0.101099
KEGG_WNT_SIGNALING_PATHWAY	146	0.30251	1.305684	0.041932	0.140282	0.102724
KEGG_FC_EPSILON_RI_SIGNALING_PATHWAY	73	-0.40375	-1.41221	0.045455	0.149351	0.109364
